# Born this way? A review of neurobiological and environmental evidence for the etiology of psychopathy

**DOI:** 10.1017/pen.2019.7

**Published:** 2019-10-23

**Authors:** Annabelle Frazier, Patricia A. Ferreira, Joseph E. Gonzales

**Affiliations:** Department of Psychology, University of Massachusetts Lowell, Lowell, MA, USA

**Keywords:** psychopathy, adversity, neurobiology of psychopathy, antisocial personality

## Abstract

Across a significant body of research, psychopathy has often been conceptualized as a biologically based malady. In this research, genetic and neurobiological differences have been conceptualized to underlie psychopathy, while affected individuals’ life experiences only influence expressed psychopathic features and their severity. Psychopathy research has largely ignored developmental evidence demonstrating significant influences of environment on both biological and behavioral processes, resulting in several prominent criticisms (Edens & Vincent, 2008; Loeber, Byrd, & Farrington, 2015). The current review was conducted with two main aims: (a) to collect and consider etiological evidence from the extant body of research on genetic and neurobiological factors in psychopathy; and (b) to evaluate findings from genetic, neurotransmitter, brain structure, and brain function studies in the context of relevant evidence from developmental research. Examples from research on adversity and traumatic stress, a common correlate of psychopathy, were used to highlight current research gaps and future directions to aid in the integration of developmental and neurobiological research agendas. While some promising evidence exists regarding possible underlying neurobiological processes of psychopathic traits, this evidence is insufficient to suggest a largely biological etiology for the disorder. Further, information from developmental and epigenetic research may suggest complex, multidimensional trajectories for individuals experiencing psychopathy. Based on these observations, the authors make several recommendations for future research, as well as for current clinical application and practice.

Psychopathy is a clinical term originating in early 19th-century psychiatry, first comprehensively described in Cleckley’s (1941) seminal book, *The Mask of Sanity*. In recent years psychopathy has been used extensively in clinical, legal, and forensic settings (Edens & Vincent, 2008; Hare, 1996, 1999). Psychopathy is often described as an untreatable personality disorder consisting of an apparent absence of empathy and remorse, along with superficial charm, shallow relationships, and rational, cold-blooded self-gratification, which often occurs at the expense of others (Hare, 1996, 1999, 2003; Moss & Prins, 2006). Today, the label can produce significant consequences within the legal realm, such as increased sentence severity and likelihood of execution (Cox, Edens, Rulseh, & Clark, 2016; Edens, Davis, Fernandez Smith, & Guy, 2013; Hare, 1996) and harsher, less engaged treatment in community settings (Edens & Vincent, 2008; Kirkman, 2008; Salekin, Worley, & Grimes, 2010; Vidal & Skeem, 2007).

In his 1999 book *Without Conscience*, developer of the Psychopathy Checklist (PCL), Robert Hare, explained that while the terms *psychopath* and *sociopath* are used interchangeably, the distinction lies in how one interprets the *origins* and *determinants* of each label. Generally, the former is used by those who ascribe the label a perdominantly biological etiology, whereas environmental factors are highlighted by those using the latter. A similar distinction is often made in considering *primary* and *secondary* psychopathy, where the former is thought to be a result of biological deficits, while the latter is attributed to various forms of social disadvantage (Newman, MacCoon, Vaughn, & Sadeh, 2005; Vaughn, Edens, Howard, & Smith, 2009).

In the following decades, Hare’s implied position – that psychopathy was much more a biological or genetic malady – has gained largely uncritical acceptance in both research and practice (Blair, 2003, 2006; Hare & Neumann, 2008). Researchers have invested heavily in exploring the various neurological and genetic factors suspected to cause or underlie psychopathic traits. The premise of such research has been that genes influence brain structures that are associated with psychopathic tendencies (Blair, 2003; Hare & Neumann, 2008). Environmental influences merely act to shape psychopathic characteristics, which manifest in childhood as callous unemotional (CU) traits, and are then stable throughout the lifespan (Blair, 2003; Hare & Neumann, 2008; Viding, Blair, Moffitt, & Plomin, 2005).

## 1. Developmental parallels

Prior to the advent of medicalized theories of its etiology, psychopathy was often regarded as environmentally derived (Blair, 2003, 2006; Hare & Neumann, 2008). In a parallel to Cleckley’s (1941) conceptualization of psychopathy, Bowlby (1944) described 14 “affectionless psychopaths,” who would meet today’s standard for diagnosis of reactive attachment disorder, given their documented behavior, affect, and histories of early and prolonged deprivation of care (Follan & Minnis, 2010). Bowlby hypothesized that deficits in early bonding with a consistent caregiver had caused these young people to perceive others as unworthy of trust, empathy, and concern (Saltaris, 2002). Subsequently, insecure attachment patterns (Frodi, Dernevik, Sepa, Philipson, & Bragesjö, 2001) and neglectful and maltreating family environments (Krischer & Sevecke, 2008; Marshall & Cooke, 1999; Piquero et al., 2012) have been reported for individuals high in psychopathic traits.

Developmental research points toward the critical influence of early caregiving in healthy emotional development. Specifically, caregiver attunement and responsiveness to an infant’s emotional and physical needs plays a critical role in many psychological and physiological development processes. For instance, Hane and Fox (2006) reported that even typical variations in maternal sensitivity and intrusiveness affected infants’ social interaction, with infants whose mothers were less sensitive and/or more intrusive showing significantly less interest in social interaction and significantly more negative affect than those whose mothers were more sensitive and less intrusive. More importantly, even in their sample of low-risk, non-maltreated children, parenting differences were associated with neurological differences among the children (Hane & Fox, 2006).

These neurological differences (Hane & Fox, 2006) appear to be the product of neurobiological changes during a critical developmental period, caused by the infant’s interaction with his or her social environment (Gerhardt, 2006; Schore, 2005). For example, Strathearn (2011) pointed out numerous biological influences of neglect. Observations of reductions in dopamine transporter binding in the ventral striatum, which cause elevated baseline dopamine levels, increased dopamine release in response to acute stress in adulthood, and increased sensitivity to psychostimulants were noted (Strathearn, 2011). In a retrospective analysis, Pruessner, Champagne, Meaney, and Dagher (2004) reported increased dopamine and cortisol release during stressful situations in individuals who reported low-quality relationships with caregivers in childhood.

Noteworthy is a timeline for the aforementioned neurobiological changes. Infants’ brain development begins with a growth spurt of the right hemisphere during the first 2 years of life (Schore, 2005). The right hemisphere – involved in emotional and social processing – maintains dominance for the first 3 years after birth, until the left hemisphere’s development catches up (Schore, 2005). Across longitudinal and cross-sectional brain imaging studies, chronic stress, deprivation, or maltreatment in the first 3 years of life has been shown to cause brain volume reductions and significant brain development abnormalities in affected 3-year-olds (Gerhardt, 2006; Perry & Pollard, 1997; Teicher & Samson, 2016). Yet children are highly unlikely to remember or have the ability to report on these early environments due to infantile amnesia (Peterson, Warren, & Short, 2011). Consequently, the impact of adversity in infancy and early childhood cannot be captured by common traumatic exposure measures.

## 2. Exploring current research on psychopathy

Currently, despite the absence of conclusive evidence of a specific innate cause for psychopathy and despite indications of significant environmental influences on its development, research continues to focus principally on the search for its biological determinants, often to the exclusion of social, developmental, and environmental factors. Such efforts have also been extended to children, with both a modified version of the Psychopathy Checklist (PCL-Youth Version) and research targeting children with CU traits, which are argued to precede psychopathy in adulthood (Barry et al., 2000; Frick, 2004; Viding et al., 2005). In these research lines, researchers hope to identify psychopathic traits when they are more malleable, or find environmental triggers that activate the expression of genetically based psychopathic traits (Barry et al., 2000; Frick, 2004; Viding et al., 2005).

The application of psychopathy to children’s underdeveloped personalities has drawn the criticism of developmental psychologists and life-course criminologists alike (Edens et al., 2013; Edens & Vincent, 2008). In their criticism of the practice, Edens and Vincent (2008) note that along with the increase in research evaluating psychopathic traits in youth, the applied (clinical and forensic) use of these measures has been on the rise. Yet, in adult populations, such applications lead to inherently negative, life-altering outcomes (Edens et al., 2013). Given limited evidence of temporal stability in personality characteristics, such applications can pose serious consequences on children whose current behavior and personality may be nothing beyond a mere state (Edens & Vincent, 2008). Notably, *heterotypic continuity*, the differential manifestation of symptoms over time (Cicchetti & Rogosch, 1996; Vitacco & Vincent, 2006), and typical developmental shifts in personality have been observed repeatedly in research (Edens & Vincent, 2008). For instance, when assessing the stability of psychopathic characteristics during the transition to adulthood, Hawes, Mulvey, Schubert, and Pardini (2014) noted a decrease in these characteristics over time. The *Diagnostic and Statistical Manual 5* (*DSM-5*, 2013), and previous editions, specifically excludes children from its diagnosis of antisocial personality disorder (APD), based on similar concerns, while allowing diagnosis with oppositional-defiant disorder and conduct disorder in youth.

These research trends have been more broadly criticized for failing to adequately integrate forensic and developmental research. For instance, Loeber, Byrd, and Farrington (2015) argued that criminological and clinical research is largely based on male inmate or patient samples, and is rarely longitudinal. Consequently, neurological differences identified in this research cannot be shown to represent life-course stable abnormalities rather than normal or temporary variants. These critiques implicate the need to integrate clinical and developmental approaches to further develop the knowledge base on the etiology of antisociality (Edens & Vincent, 2008; Loeber et al., 2015). However, few comprehensive efforts for such an integration have been made. The purpose of this article is to offer one such effort, by (a) evaluating the degree of integration of environmental and developmental correlates in biological research and (b) reviewing neurobiological research findings in the context of their developmental correlates. Conclusions regarding both the current state of knowledge on the etiology, treatment, and prognosis of psychopathy are also discussed.

## 3. Method

### 3.1. Inclusion criteria

Since 1999, when Hare’s book was published, a multitude of studies have investigated psychopathic traits in biological terms. However, much of this literature has been affected by two areas of debate among scholars and practitioners: (a) whether the psychopathy construct is driven by violent or rule-breaking behavior (Blair, 2001; Porter & Woodworth, 2006; Skeem & Cooke, 2010) and (b) whether APD and psychopathy represent two distinct constructs (Ogloff, 2006; Patrick & Brislin, 2014). Within this body of literature, violence and aggression, CU traits, and antisocial personality and behavior are often discussed as proxies to (or precursers of) psychopathy (Barry et al., 2000; Beaver et al., 2013; Blair, 2001; Gunter, Vaughn, & Philibert, 2010; Hoenicka et al., 2007; Hyde et al., 2014; Raine et al., 2003; Rautiainen et al., 2016). The authors therefore elected to consider studies that measured these psychopathy traits using established measures of the construct (e.g., PCL and PCL-R), but also theoretically related traits and behaviors used in lieu of psychopathy measures due to the age of the subjects (e.g., CU traits) or their role as a proto-psychopathy indicator (e.g., antisocial behavior).

Within the current review, authors sought to identify studies that (a) were published between 1999 and 2017; (b) described quantitative analyses that included some measure of psychopathic tendency (broadly construed to include antisocial personality and behavior); and (c) included some neurobiological (i.e., genetic, neurotransmitter, brain imaging) measures with a human sample.

### 3.2. Search procedure

Based on the aforementioned criteria, the authors conducted a literature search on Google Scholar, using keyword combinations to account for the largest possible body of research in psychopathic and antisocial traits. For example, keywords included “psychopathy+gene,” as well as “antisocial+personality+neurotransmitter.” Abstracts of studies identified by this search (>1500) were then reviewed for relevance to the current review. Studies that (a) did not include a neurobiological measure, (b) where no human sample was used, (c) in which only traits or behaviors indirectly related to psychopathy were assessed (e.g., criminal or delinquent behavior; sexual aggression), or (d) where no empirical data collection was reported (i.e., theoretical papers and literature reviews) were excluded from this review. This process resulted in the inclusion of 54 studies in this review. Upon detailed review, four additional studies were excluded because they used the same dataset for multiple similar analyses (National Longitudinal Study of Adolescent Health; sample from employment temp agencies) or because characteristics of the sample were not described, ultimately resulting in a review of 50 studies.

### 3.3. Initial evaluation of studies

Each study was then assessed to determine whether at least one environmental factor (e.g., socioeconomic status, maltreatment, or trauma) was analyzed. The frequency with which environmental/developmental factors were reported was counted. Of the 50 studies, 17 (34%) included at least one environmental covariate or control. The list of studies included, and factors evaluated in them, is presented in Table 1. Similarly, studies were evaluated for their use of PCL type instruments and use of forensic and clinical samples, adults and children, and male and female participants.

**Table 1.** Characteristics of the reviewed studies

Study	Focus	Environmental/developmental factors	Psychopathy checklist	Forensic/clinical sample	Adults	Males and females
Yang, Raine, Narr, Colletti, and Toga (2009)	Brain volume/activation	Yes	Yes	No	Yes	Yes
Yang, Raine, Colletti, Toga, and Narr (2010)	Brain volume/activation	Yes	Yes	No	Yes	Yes
Marsh et al. (2013)	Brain volume/activation	No	Yes	Yes	No	No
Pardini, Raine, Erickson, and Loeber (2014)	Brain volume/activation	No	No	No	Yes	No
Boccardi et al. (2011)	Brain volume/activation	No	Yes	Yes	Yes	No
Jones, Laurens, Herba, Barker, and Viding (2009)	Brain volume/activation	No	No	No	No	No
Marsh et al. (2011)	Brain volume/activation	No	Yes	No	No	Yes
White et al. (2012b)	Brain volume/activation	No	No	No	No	Yes
Birbaumer et al. (2005)	Brain volume/activation	No	Yes	Yes	Yes	No
Hyde, Byrd, Votruba-Drzal, Hariri, and Manuck (2014)	Brain volume/activation	No	No	Yes	Yes	Yes
Raine, Lencz, Bihrle, LaCasse, and Colletti (2000)	Brain volume/activation	Yes	Yes	No	Yes	No
Ermer, Cope, Nyalakanti, Calhoun, and Kiehl (2012)	Brain volume/activation	No	Yes	Yes	Yes	No
de Oliveira-Souza et al. (2008)	Brain volume/activation	No	Yes	Yes	Yes	Yes
Raine et al. (2003)	Brain volume/activation	Yes	Yes	No	Yes	No
Soderstrom et al. (2002)	Brain volume/activation	No	Yes	Yes	Yes	Yes
Laakso et al. (2001)	Brain volume/activation	No	Yes	Yes	Yes	No
Müller et al. (2003)	Brain volume/activation	No	Yes	Yes	Yes	No
Rilling et al. (2007)	Brain volume/activation	No	No	No	Yes	Yes
Barkataki, Kumari, Das, Taylor, and Sharma (2006)	Brain volume/activation	No	No	Yes	Yes	No
Kiehl et al. (2004)	Brain volume/activation	No	Yes	Yes	Yes	No
Longato-Stadler, af Klinteberg, Garpenstrand, Oreland, and Hallman (2002)	Genetics	No	Yes	Yes	Yes	No
Chen et al. (2005)	Genetics	Yes	No	No	No	No
Young et al. (2006)	Genetics	Yes	No	No	No	Yes
Hoenicka et al. (2007)	Genetics	No	No	Yes	No	Yes
Ponce et al. (2008)	Genetics	No	No	Yes	No	No
Williams et al. (2009)	Genetics	No	No	Yes	No	Yes
Fowler et al. (2009)	Genetics	Yes	No	Yes	Yes	No
Garcia, Aluja, Fibla, Cuevas, and García (2010)	Genetics	No	No	Yes	Yes	No
Sadeh et al. experiment I (2010)	Genetics	Yes	No	Yes	Yes	No
Sadeh et al. experiment II (2010)	Genetics	Yes	No	Yes	Yes	No
Basoglu et al. (2011)	Genetics	No	Yes	Yes	Yes	No
Wu and Barnes (2013)	Genetics	Yes	No	Yes	Yes	No
Sadeh, Javdani, and Verona (2013)	Genetics	Yes	No	Yes	Yes	Yes
Beaver et al. (2013)	Genetics	No	No	Yes	No	Yes
Van de Giessen et al. (2014)	Genetics	No	No	No	No	Yes
Dadds et al. (2014)	Genetics	Yes	No	Yes	Yes	No
Kolla et al. (2015)	Genetics	No	Yes	Yes	Yes	Yes
Rautiainen et al. (2016)	Genetics	No	Yes	No	Yes	Yes
Caspi et al. (2002)	Genetics	Yes	Yes	Yes	No	Yes
Hallikainen et al. (1999)	Genetics	No	Yes	Yes	No	No
Dolan and Anderson (2003)	Neurotransmitter	No	No	No	Yes	No
da Cunha-Bang et al. (2016)	Neurotransmitter	Yes	No	Yes	Yes	No
Buckholtz et al. (2010)	Neurotransmitter	Yes	Yes	Yes	Yes	No
Fanning, Berman, Guillot, Marsic, and McCloskey (2014)	Neurotransmitter	No	No	No	Yes	Yes
Stanley et. al. (2000)	Neurotransmitter	No	No	Yes	Yes	No
Moul, Dobson-Stone, Brennan, Hawes, and Dadds (2013)	Neurotransmitter	Yes	Yes	Yes	Yes	Yes
Soderstrom, Blennow, Sjodin, and Forsman (2003)	Neurotransmitter	No	No	Yes	No	No
Rosell et al. (2010)	Neurotransmitter	No	No	No	Yes	Yes
Moore, Scarpa and Raine (2002)	Neurotransmitter	Yes	Yes	Yes	Yes	Yes
Gerra et al. (2003)	Neurotransmitter	No	No	Yes	Yes	Yes

## 4. Sampling and measurement trends

Several general trends were observed in reviewed studies. First and foremost, environmental and developmental factors were not adequately integrated with biological research. Sixty-six percent of the studies reviewed did not consider any influences of environment or relevant developmental processes. In the remaining studies, the most common environmental variables included socioeconomic status (10% of studies), family environment (8% of studies), and maltreatment (8% of studies). In these studies, divergent measures and definitions made comparisons across findings difficult to make.

Second, the use of PCL-type measures – e.g., PCL-Revised (PCL-R, Hare, 1991, 2003) and PCL-Screening Version (PCL-SV, Hart, Cox, & Hare, 1995) – decreased over time, corresponding with an increase in the use of child samples (16% of studies), although adults remained the primary subjects of this research. Overall, 46% of studies used PCL instruments, while 54% gauged psychopathic tendencies in other ways. The latter studies predominantly used the Structured Clinical Interview for DSM (SCID) diagnosis (12% of studies) along with some measure(s) of criminality or aggression – though others used versions of the Antisocial Process Screening Device (8% of studies), the Psychopathic Personality Inventory (6% of studies), and the NEO Five-Factor Inventory (4% of studies). While the majority of these instruments correlate with one another, they measure psychopathy differently (e.g., informal interview compared to self-report), and many consider criminality, which may be related to – but not essential to – the psychopathy construct. Studies that utilized PCL-type measures were similar to studies using other instruments across demographic characteristics (i.e., inclusion of female participants), except that 83% used adult forensic or prisoner samples. Given that the PCL-R requires a record review as part of accurate administration (Hare, 2003), reliance on forensic samples in which these records exist for research using the PCL-R is unsurprising.

Further, neurobiological studies consistently favored male participants over females, and both forensic and clinical samples over community samples (36% of studies). Most frequently, adult participants were recruited from prisons and forensic institutional settings (26% of studies). Yet the influence of residing in the institution (Boxer, Middlemass, & Delorenzo, 2009; Bukstel & Kilmann, 1980; Haney, 2003; Wooldredge, 1999) was not itself considered in the research. Female participants were included in 44% of studies, but in these studies, 50 male participants were recruited for every 27 female participants, on average. None of the studies used a female-only sample, despite concerns regarding the capacity of psychopathy measures to adequately capture the construct in females (Vitale, Smith, Brinkley, & Newman, 2002). Relatedly, only 30% of studies considered nonwhite racial and ethnic groups. While all humans share basic biological characteristics, genetic and biomedical differences (and thus risk factors) within different ethnic groups have been noted (Burchard et al., 2003), including different genetic correlates of antisociality (Lu, Lin, Lee, Ko, & Shih, 2003). Importantly, these shortages in participant diversity pose significant limitations to the generalizability of psychopathy research findings to the population.

## 5. Genetic etiology

Several authors have argued for a genetic or hereditary pathway toward psychopathy (Auty, Farrington, & Coid, 2015; Blair, 2003, 2006; Viding et al., 2005). The notion is that psychopathic individuals inherit a genetic makeup that manifests in altered brain functioning and physiological reactivity, and along with some environmental triggers or influences, shapes their behavior in childhood (Blair, 2003, 2006; Viding et al., 2005). For instance, Viding et al. (2005) reported finding strong evidence of heredity and no evidence of shared environmental influences in their study of 7-year-old twins with antisocial behavior and CU traits. Similarly, Auty et al. (2015) found strong evidence of transmission of psychopathy from fathers to their children, though this was mediated by environmental factors. Given the strength of genetic influence reported in these and similar findings, examining the evidence for specific genetic factors is important.

### 5.1. MAO-A

The strongest cumulative evidence base for a genetic pathway toward psychopathy is associated with the low-expression variant of the *Monoamine Oxidase-A* (*MAO-A*) gene, which encodes an enzyme that degrades mono-amine neurotransmitters – that is, dopamine, norepinephrine, and serotonin (Caspi et al., 2002; Cicchetti, Rogosch, & Thibodeau, 2012). The low-expression variant of the *MAO-A* gene is linked to the X chromosome. Possessing only a single X chromosome, males are more likely to be influenced by a low-expression variant (Hunter, 2010).

In all eight studies investigating *MAO-A* polymorphisms (Beaver et al., 2013; Caspi et al., 2002; Fowler et al., 2009; Kolla et al., 2015; Longato-Stadler et al., 2002; Sadeh et al., 2013; Williams et al., 2009; Young et al., 2006), statistically significant correlations were identified between the short allele and psychopathic and/or antisocial traits. The use of differing measures of psychopathy and antisocial behavior, along with inadequate delineation of participants’ histories of criminality and delinquency from the psychopathy construct, posed a significant limitation for many studies. Several authors found associations between subjects’ criminality and a lower-expression variant of the *MAO-A* gene (Beaver et al., 2013; Kolla et al., 2015; Sadeh et al., 2013), or specifically investigated APD diagnosis (Longato-Stadler et al., 2002; Young et al., 2006), which placed significantly more weight on rule-breaking behavior (Hare, 1996). Focusing specifically on core psychopathic traits, only two studies (Kolla et al., 2015; Williams et al., 2009) identified significant differences between carriers of lower- and higher-expression *MAO-A* variants (i.e., increased psychopathic traits in short allele carriers). Fowler et al. (2009) found lower-expression *MAO-A* variants to be significantly related to emotional dysfunction scores in youth diagnosed with attention deficit hyperactivity disorder (ADHD), but the cumulative effect size for the same relation with total psychopathy-related scores was medium-sized (*f*^2^ = 0.16).

In 2002, Caspi et al. observed an interaction between childhood adversity and the MAO-A risk allele, demonstrating that for those who carried this allele, adversity was positively related with psychopathy. Building on findings from Caspi et al. (2002), several subsequent MAO-A studies (Sadeh et al., 2013; Williams et al., 2009; Young et al., 2006) included measures of childhood adversity or maltreatment. However, whereas some studies found associations between maltreatment, adversity, and rule-breaking behavior (Sadeh et al., 2013; Young et al., 2006) or between the risk allele and antisocial behavior in participants who experienced severe family adversity (Fowler et al., 2009), others did not (Williams et al., 2009). Notably, these studies varied not only in their measures of psychopathic traits but also in their measurement of adversity and maltreatment. For instance, Williams et al.’s (2009) analysis used only a dichotomous adversity score, comparing participants who experienced more than three adverse events to those who experienced three or fewer. Nonetheless, the interaction between MAO-A and maltreatment observed by Caspi et al. (2002) was not replicated in these subsequent studies.

### 5.2. 5-HTT

Based on observations of reduced serotonin in aggressive and impulsive individuals (Ferguson & Beaver, 2009; Goodman & New, 2000; Lesch & Merschdorf, 2000), multiple studies have sought to associate 5-HTT with psychopathy. Findings in the seven studies that examined this relationship (Fowler et al., 2009; Garcia et al., 2010; Hallikainen et al., 1999; Sadeh et al., 2010, experiments 1 and 2; Sadeh et al., 2013; Van de Giessen et al., 2014) were inconsistent. Sadeh et al. (2013), for instance, found that PCL:SV Factor 2 scores were significantly related to carrying the long allele of 5-HTT and to Childhood Trauma Questionnaire scores, though no interaction was identified. Similarly, in Sadeh et al. (2010, study II), CU traits increased in long/long allele carriers, but only as socioeconomic resources decreased. In contrast, aggression, impulsivity, and antisocial behavior was found to be related to carrying the short allele of 5-HTT (Garcia et al., 2010; Sadeh et al., 2010, study I). No relation between aggression and 5-HTT availability was identified in Van de Giessen et al. (2014), but the authors observed a positive relation with callousness traits.

### 5.3. Dopamine receptor genes

Because of their role in the pleasure/reward system in humans, dopaminergic system genes have been a source of considerable research relating to violence, aggression, and antisocial behavior (Ferguson & Beaver, 2009; Ferguson, 2010). Nonetheless, only three of the studies investigated dopaminergic gene systems (Hoenicka et al., 2007; Ponce et al., 2008; Wu & Barnes, 2013). An additional study (Fowler et al., 2009) investigated the *COMT* gene, which regulates the production of the dopamine degrading enzyme, and is therefore discussed here as well.

All four studies reported relatively small impact of dopaminergic system genes. For instance, Fowler et al. (2009) reported the high-activity *COMT* genotype was statistically associated with significantly higher emotional dysfunction scores, with a small effect size (*f*^*2*^ = 0.08), and without significant impact on total psychopathy scores. Wu and Barnes (2013) found that only DRD4 (and not DAT1 or DRD2) was significantly related to psychopathic personality traits. Yet carrying two DRD4 risk alleles (as compared to zero) increased participants’ mean psychopathy scores by only two points (from 55.97 to 57.99, on a scale of 23–115). Hoenicka et al. (2007) and Ponce et al. (2008) found *DRD2* gene polymorphisms to be associated with higher scores on the International Personality Disorder Examination (IPDE). Hoenicka et al. (2007) reported these genetic markers (when combined) may be responsible for 11.4% of the variance in psychopathy scores.

### 5.4. Other targets

Several other genetic polymorphisms have been analyzed for associations with psychopathy-related tendencies. These included *SNAP25* t-snare protein gene (Basoglu et al., 2011; Hoenicka et al., 2007), *OXT*, the gene responsible for oxytocin production (Dadds et al., 2014), and the CNR1 and FAAH cannabinoid receptor gene polymorphisms (Hoenicka et al., 2007). Hoenicka et al. (2007) found that variants of FAAH and CNR1 were related to an increase in PCL-R scores for their subjects, but found no impact of *SNAP25* genes. In contrast, Basoglu et al. (2011) reported that a variant of the SNAP25 gene was much more common in antisocial personality subjects, and that these antisocial subjects had significantly higher novelty-seeking scores. Dadds et al. (2014) observed maturation-related differences in genetic activity, with the low-CU group showing lower methylation of the *OXT* gene in older children, while the opposite trend was observed in the high-CU group. In contrast, Rautiainen et al.’s (2016) genome-wide association study found none of these associations in their subjects, instead reporting an observed association between APD diagnosis (based on SCID-II interview) and Human Leukocyte Antigen (HLA) system genes, which impact the immune system and express in the brain.

### 5.5. Reconsidering gene-environment interaction

Overall, it is well established that experiences and environmental influences can moderate the effects of individual polymorphisms on behavior and impact biological pathways that intersect with genetic influences, ultimately affecting gene expression (Manuck & McCaffery, 2014). The latter has been repeatedly shown to occur in maltreated children (Manuck & McCaffery, 2014; McDade et al., 2017), with children who experienced early stress showing different gene methylation, and thus expression, in adolescence than those whose environments were relatively low in stress (McDade et al., 2017; Essex et al., 2013). Despite this, fewer than 50% of genetic studies included any environmental covariates, and none accounted for epigenetic change. Epigenetic mechanisms are rarely considered in relation to psychopathic trait development but may provide important insights regarding its etiology (Gillett & Tamatea, 2012).

While the aforementioned findings regarding genetic factors in psychopathy are variable, their interpretation is further complicated by gene-environment interaction research in antisocial personality traits (Cicchetti et al., 2012; Larsson et al., 2008). Particularly, maltreatment and childhood adversity have been linked to increases in CU or APD traits (Beach, Brody, Todorov, Gunter, & Philibert, 2010; Caspi et al., 2002; Chapman, Dube, & Anda, 2007; Cicchetti et al., 2012; Larsson et al., 2008). Similarly, Kolla et al. (2015) reported a higher prevalence of childhood physical abuse in offenders exhibiting psychopathic traits, and that these early experiences were associated with increases in reactive aggression. Finally, several investigations (Hoenicka et al., 2007; Ponce et al., 2008) have focused on psychopathy in those with substance use histories, and these have offered further support for the impact of childhood adversity on later substance use (Anda et al., 2002; Dube et al., 2003).

Other behavioral and environmental factors may influence gene expression in ways that genetic research has yet to predict. For instance, several of the same genetic polymorphisms have been investigated in both psychopathy and ADHD research. Indeed, subjects with ADHD were the focus of Fowler et al (2009). However, childhood ADHD is associated with significant problems in social, school, and emotional functioning (DuPaul, McGoey, Eckert, & VanBrakle, 2001). These impairments have been linked with problematic family functioning, including greater family stress, higher rates of parental psychopathology, and conflicted parent–child relationships (Deault, 2010), which may produce a developmental cascade unaccounted for by currently available research.

## 6. The role of neurotransmitters

Along with genetic polymorphism examinations, some researchers have sought to identify neurotransmitter involvement in psychopathy (Glenn & Raine, 2008; Wu & Barnes, 2013). This focus is not dissimilar to the interest in neurotransmitter activity underlying other mental disorders, based on the neurochemical imbalance hypothesis, which suggests that an imbalance in the brain chemistry of affected individuals leads to a variety of mental health problems (Deacon, 2013; France, Lysaker, & Robinson, 2007).

### 6.1. Norepinephrine

Often known as noradrenaline, norepinephrine functions as an activation neurotransmitter in the threat-response system. It has been long hypothesized to interact with dopamine in a system of neurochemicals linked to reward-oriented behavior and response to stress, ultimately leading to undesirable or dysfunctional behavior such as aggression or absent empathy (Antelman & Caggiula, 1977; Cases et al., 1995; Thomas & Palmiter, 1997). Of the studies included in this review, the only one to investigate norepinephrine (Gerra et al., 2003) did not find a significant relationship with psychopathy.

### 6.2. Serotonin

Researchers have long speculated about an association between core psychopathic traits and nonoptimal stability and efficiency in serotonin functioning, whereby healthy levels of baseline arousal dampen psychophysiological responses to threat, punishment, and social stimuli (Yildirim & Derksen, 2013). These hypotheses have been supported by the finding that serotonin inhibits aggressive behavior (Carrillo, Ricci, Coppersmith, & Melloni, 2009; Cases et al., 1995; de Boer & Koolhaas, 2005). Serotonin was, therefore, the focus of a significant number of recent studies on antisocial and psychopathic samples (da Cunha-Bang et al., 2016; Dolan & Anderson, 2003; Fanning et al., 2014; Gerra et al., 2003; Moore et al., 2002; Moul et al., 2013; Rosell et al., 2010; Soderstrom et al., 2003; Stanley et al., 2000). In the eight reviewed studies, lower serotonin levels were linked more often to increased impulsivity traits (da Cunha-Bang et al., 2016; Dolan & Anderson, 2003; Moore et al., 2002) than to callous-unemotional traits (Moul et al., 2013) or aggression (Stanley et al., 2000). A deficiency in serotonin production may, therefore, not actually underlie aggressive, antisocial tendencies but rather impulse control problems that may lead to antisocial behaviors. Notably, Moore et al. (2002) reported that this effect was moderated by age, thus supporting the notion that differences in serotonin production are not life-course stable.

### 6.3. Dopamine

Due to its association with hyperactivity, aggressive, and reward-motivated behavior, dopamine has been investigated in three of the reviewed studies (Buckholtz et al., 2010; Gerra et al., 2003; Soderstrom et al., 2003). It is often assumed that dopaminergic genes cause an overabundance of the neurotransmitter, which produce some of the behavioral and emotional manifestations common in psychopathy. However, results of these studies have contradicted this idea. For instance, Gerra et al. (2003) found that subjects with antisocial personalities showed reduced dopaminergic receptor sensitivity, suggesting that their subjects’s brains were less responsive to dopamine and thus required more of it. In contrast, Buckholtz et al. (2010) reported dopamine system hyperactivity in their subjects, which they associated with antisocial-related impulsivity. Soderstrom et al. (2003) found that aggression was related to a high dopamine turnover together with serotonergic dysregulation. Since not all psychopathic individuals are both aggressive and impulsive (i.e., some may only possess one of the two traits), these latter findings suggest that researchers may be observing correlates of aggression and impulsivity rather than psychopathy.

## 7. Neurological differences

Observations of the emotional and empathic deficits in psychopathic offenders have led to hypotheses of functional and/or structural neurological abnormalities in antisocial individuals (Shamay-Tsoory, Harari, Aharon-Peretz, & Levkovitz, 2010; Yang et al., 2009). Specifically, it has been suggested that, as a result of inherited genetic differences affecting neurotransmission and neurodevelopment, psychopathic individuals have decreased activation and volume in key areas of the brain, including the orbitofrontal cortex (Mitchell, Colledge, Leonard, & Blair, 2002) and amygdalae, as well as broader gray matter volume reductions (Blair, 2006; Blair, Peschardt, Budhani, Mitchell, & Pine, 2006).

### 7.1. Whole-brain studies

Multiple whole-brain volume (Barkataki et al., 2006; Raine et al., 2003), blood flow (Soderstrom et al., 2002), and neural activation (Marsh et al., 2013; Müller et al., 2003; Rilling et al., 2007) studies assessed differences between typical and psychopathic individuals. Of the studies investigating whole-brain volume, one observed increased volume (Raine et al., 2003), while the other observed decreased volume (Barkataki et al., 2006). Oddly, both studies included participants with schizophrenia symptoms. Barkataki et al. (2006), whose sample comprised 50% schizophrenia-affected participants, reported significant differences in intelligence between them and antisocial participants (who did not exhibit similar psychotic symptoms), along with reduced brain volume in comparison to nonviolent individuals and healthy controls. In contrast, Raine et al. (2003) reported that though the difference between participants and controls in psychotic symptoms was statistically significant (i.e., 40% met schizophrenia spectrum diagnosis criteria), they believed these symptoms did not significantly affect their participants’ outcomes on the measures of interest, reporting increased volume as compared to controls. Yet such a conclusion – that the presence of psychosis would not be meaningful for a brain volume analysis of antisocial participants – is at odds with strong evidence for brain volume and function differences in psychotic individuals (Radua et al., 2012). Further, the presence of psychotic symptoms is not typical or expected for antisocial or psychopathic individuals.

Regionally, Laakso et al. (2001) observed a positive correlation between the hippocampal volumes and age of the subjects, while also noting negative correlations between regional volume reductions and PCL-R Factor 1 scores. In analyzing blood circulation in the brain, Soderstrom et al. (2002) reported no significant correlation between bloodflow and total PCL-R scores, though strong negative correlations were identified between the temporal blood flow and Factor 1 (emotional/affective) PCL-R scores, with a Spearman’s rho of .34 for the right, and .49 for the left side. The highest-scoring subjects exhibited significantly lower bloodflow in the head of the caudate nucleus, the hippocampus, the left thalamus, the amygdala, and the medial and lateral frontal areas (Soderstrom et al., 2002).

Several studies have reported different activation patterns across a variety of tasks in the brains of individuals with psychopathic traits compared to those of controls. Specifically, reduced activation in areas influencing autonomic regulation (i.e., the rostral anterior cingulate cortex and right ventral striatum) in response to perceived personal distress or pain, and in the affective regulation areas (i.e., the amygdala and insula) in response to others’ fear, distress and pain, has been reported in psychopathic individuals (Marsh et al., 2013). Similarly, reduced activation was found in areas responsible for reinforcement learning (i.e., the dorsal striatal circuits; Marsh et al., 2013; Rilling et al., 2007).

Like Birbaumer et al.’s (2005), Marsh et al. (2013) subjects showed significantly less activation in the left amygdala, left middle and right anterior insula, anterior cingulate, right secondary somatosensory cortex, and the right ventromedial orbitofrontal cortex in the second half of the acquisition phase of the stimulus learning task. In contrast, Kiehl et al. (2004) reported that activation (in response to stimulus words) was overall similar between subjects and controls, except in the right anterior superior temporal gyrus for psychopathic individuals’ responses between abstract and baseline phases (Kiehl et al., 2004). Further, Kiehl et al.’s (2004) subjects also exhibited reduced activation in these regions in response to concrete stimuli.

In a prisoners’ dilemma game, male (but not female) subjects higher in psychopathy showed reduced activation in the right amygdala after a partner defected, and reduced activation within the rostral anterior cingulate cortex and dorsolateral prefrontal cortex when choosing to defect (Rilling et al., 2007). In response to the International Standardized Affective Picture System positive/negative emotion task, subjects with higher psychopathy scores showed increased right-sided activation in prefrontal regions, anterior cingulate, and the amygdala for negative emotions, and reduced activation in the right subgenual cingulate and right medial temporal gyrus, left lobulus paracentralis, left dorsal cingulate, and left parahippocampal gyrus (Müller et al., 2003). In contrast, positive emotions induced increased activation in the left gyrus frontalis, and right medial frontal and right medial temporal gyrus, leading to the conclusion that psychopathic individuals’ emotional responsivity differed from that of other subjects (Müller et al., 2003).

### 7.2. Gray matter

Multiple research papers (de Oliveira-Souza et al., 2008; Ermer et al., 2012; Raine et al., 2000; Yang et al., 2010) have reported observations of reduced gray matter volume in the orbitofrontal cortex of psychopathic individuals. Yang et al. (2010), in comparing “successful” and “unsuccessful” psychopaths, reported that these (and other observed differences) applied only to the latter due to arrest histories. In addition, de Oliveira-Souza et al. (2008) observed gray matter volume reductions in the mid-anterior insula, and left anterior temporal cortex, while Ermer et al. (2012) noted gray matter volume reductions in the parahippocampal cortex, though these were not statistically significant in whole-brain analyses. Ermer et al. (2012) speculated that differences in gray matter in psychopathy are subtle and widespread, leading to minimal differentiation when whole-brain analyses are used. Raine et al. (2000) noted that observed reductions amounted to 11% compared to controls. These studies suggest that higher PCL-R scores may be associated with subtle reductions in gray matter volume across several paralimbic and limbic areas, implicated in emotion processing, theory-of-mind-related tasks, regulation, and behavioral control. However, Yang et al.’s (2010) distinction between individuals who have been arrested is important in interpreting these results given that the majority of psychopathy studies used forensic and institutional samples (Birbaumer et al., 2005; de Oliveira-Souza et al., 2008; Ermer et al., 2012; Kiehl et al., 2004; Laakso et al., 2001; Marsh et al., 2013; Müller et al., 2003; Soderstrom et al., 2002) – that is, these differences may not be evident in individuals with higher PCL-R scores but who avoid incarceration.

### 7.3. The amygdala

Due to its association with emotional functioning, the amygdala has been a popular focus in neurobiological studies (Boccardi et al., 2011; Hyde et al., 2014; Jones et al., 2009; Marsh et al., 2011; Pardini et al., 2014; White et al., 2012b; Yang et al., 2009). In studies that evaluated amygdala volume, one reported volume reductions (Yang et al., 2009), one reported increases (Boccardi et al., 2011), and yet another reported no difference (Pardini et al., 2014).

In observing amygdala activation patterns, right amygdala activation has been noted to increase in response to fearful faces (Jones et al., 2009), but decrease while comparing words related to legal and illegal acts (Marsh et al., 2011). Yet White et al. (2012b) reported that during low attentional load trials subjects’ amygdalae activation increased less than that of controls, as compared to high attentional load activation. Intrestingly, Hyde et al. (2014) reported that when considered separately, APD and psychopathy traits did not have a significant relation to amygdala activation. Only when both were considered together did an association emerge. Hyde et al. (2014) attributed this to suppression effects, whereby APD and psychopathy measures served as a “cooperative suppressor” for each other (Paulhus, Robins, Trzesniewski, & Tracy, 2004), amplifying the effects of the other on amygdala activation. Thus, Hyde et al. (2014) argued that one of the two constructs (antisocial personality or psychopathy) was unrelated to amygdala activation, yet raised the impact of the other on the predictive ability of the model as a whole (Cohen, Cohen, West, & Aiken, 2013; Paulhus et al., 2004).

### 7.4. Integrating environmental influences into brain research

Environmental covariates were rarely considered in brain research. Only 4 of the aforementioned 20 studies included such variables. Yet, developmental research has consistently shown that neglected, maltreated, or stress-exposed children exhibit many of the same brain abnormalities observed in in these studies. For instance, smaller left amygdala volumes and significant whole-brain gray matter reductions have been reported in post-institutionalized children (Mehta et al., 2009), as well as localized hippocampal, insular, orbitofrontal cortex, anterior cingulate gyrus, and caudate volume reductions (Dannlowski et al., 2012). Perry (2002) reported abnormal, atypical frontal-occipital circumference development of children who were chronically neglected. Though multiple studies have found increased amygdala responsivity to acute stress in people who have experienced significant early-life environmental stress or adversity (Maheu et al., 2010; Swartz, Williamson, & Hariri, 2015; White et al., 2012a), others have reported decreased amygdala activation in individuals resilient to post-traumatic stress disorder (Phan, Britton, Taylor, Fig, & Liberzon, 2006), and reciprocal and opposing relations between activation in the amygdala and medial prefrontal cortex (Shin et al., 2004).

In a first-of-its-kind recent study, Sethi et al. (2018) distinguished among psychopathic individuals with high anxiety and maltreatment (who may be classified with “secondary” psychopathy), and those without adversity or anxiety (i.e., with “primary” psychopathy). Despite similar interpersonal/affective facet scores among the two groups, the researchers indeed found differences among them in both fear-related brain activation patterns, and in amygdala activation (which was reportedly typical in those with maltreatment histories). This observation led the Sethi et al. (2018) to speculate that abnormalities in fear conditioning may play a role in the development of Factor 1 traits in the presence of anxiety, possibly in response to environmental trauma.

## 8. Psychophysiological manifestations

Several psychophysiological differences have been noted in biological research. Yildirim and Derksen (2013) proposed that these result from neurological and neurotransmitter activity differences that produce typical baseline arousal levels while dampening arousal during stressful, frightening, or adverse social experiences. Indeed, Raine et al. (2000) reported that during a social stressor task, antisocial individuals exhibited reduced autonomic activity, skin conductance, and heart rate. In a meta-analysis of 95 studies, Lorber (2004) found that low resting electrodermal activity (EDA) and low task EDA were associated with psychopathy, and that psychopathy was negatively associated with EDA reactivity. However, Lorber’s (2004) analysis did not identify a significant effect for resting heart-rate or heart-rate reactivity when aggression was considered separately from the psychopathy construct.

In studying antisocial behavior, Portnoy and Farrington (2015) found that across 114 studies, antisocial behavior was related to low resting heart rate, with no moderating impact of age, sex, study design. However, Portnoy and Farrington’s (2015) meta-analysis demonstrated that across measurements of antisociality (e.g., offending behavior, violence, aggression, psychopathy) studies differed significantly in their findings. Similarly, in a sample of Asian “primary” and “secondary” psychopathic women, the latter exhibited much higher heart rate variability across rest and stress tasks, despite significant difference in interpersonal/affective facet scores in both groups (Goulter, Kimonis, Denson, & Begg, 2019). In a sample of juveniles, a lower blink rate was observed among those labeled with “primary” (as compared to “secondary”) psychopathy (Kimonis, Fanti, Goulter, & Hall, 2017). Lorber (2004) suggested that contradictory findings may be attributable to heterogeneity in the behavioral construct (e.g., “successful” vs. “unsuccessful”; Yang et al., 2010). Indeed, some have suggested that the construct includes so many diverse presentations that a multitude of etiological pathways should be considered (Brinkley, Newman, Widiger, & Lynam, 2004). A further consideration of this multiple-pathway hypothesis can benefit from integration of biological findings with developmental literature.

### 8.1. Physiology and stress

Related to the documented neurological and neurochemical changes in stress-exposed children, alterations in physiological manifestation have been documented as well. The physiological consequences of these neurological changes are similarly diverse, with individuals’ affected traumatic stress symptoms exhibiting increased ANS activation during acute stress, while others exhibit reduced activation along with fewer PTSD symptoms (Perry, 1994, 1999; Scheeringa, Zeanah, Myers, & Putnam, 2004). These findings have prompted speculation that adaptive dissociative states may stabilize into traits in certain instances (Perry, Pollard, Blakley, Baker, & Vigilante, 1995).

## 9. Making sense of neurobiological findings

While overarching conclusions on the neuobiological mechanisms underlying psychopathic traits cannot be made due to contradictory findings, a theoretical trend emerges from the explored evidence. Suppressed activation has been noted across genetic, neurological, and psychophysiological findings. As for genetic evidence, the low-expression *MAO-A* variant is understood as having an *inhibitory* effect on the enzyme encoded by it (Caspi et al., 2002; Cicchetti et al., 2012). Although the exact nature of its influence is unclear (Sadeh et al., 2010, 2013), the *low-activity* serotonin transporter (5-HTT) variant has also been linked with antisociality (Fowler et al., 2009). As for neurological studies, many have reported a comparative *suppression* of neural activity and/or volume in designated brain structures like the orbitofrontal cortex, frontal lobes, and amygdalae (Blair, 2006; Blair et al., 2006; Mitchell et al., 2002; Raine et al., 2001). Moreover, in considering psychophysiological evidence, a pattern of *repressed* activity and/or physical responses is also pertinent, as evidenced in studies revealing depressed skin conductance and heart rate in antisocial participants (Lorber, 2004; Portnoy & Farrington, 2015; Raine et al., 2000).

Despite the noted trend, many findings challenge the proposed pattern of suppressed activation. For this reason, these observations allow for little in the way of definitive conclusions. For instance, a link between a *high-activity COMT* variant and higher “emotional dysfunction” has been found (Fowler et al., 2009). A *higher-activity* 5-HTT variant has also been shown to exert greater influence on affective-interpersonal traits when compared to its lower-activity counterpart (Sadeh et al., 2013). In addition, *higher* activation of certain brain structures (see Müller et al., 2003) has been associated with emotional reactions to both negative and positive stimuli in psychopathic subjects.

When it comes to neurotransmitter evidence, applying the suppressed activation theory becomes an even greater challenge. If the low-expression *MAO-A* variant encodes a lower-activity enzyme that reduces the breakdown of monoamine neurotransmitters, it is reasonable to theorize that this may result in increased synaptic presence of the same neurotransmitters (Cases et al., 1995; Fowler et al., 2009). However, while increased presence (or decreased degradation) of neurotransmitters appears to contradict the overarching pattern of suppression, the absolute or partial inhibitory effects associated with some of these neurotransmitters (e.g., *absent* empathy, or *decreased* psychophysiological responses; Cases et al., 1995; Thomas & Palmiter, 1997; Yildirim & Derksen, 2013) are in line with a suppression pattern.

One of the greatest challenges in drawing any trend-like conclusions concerning neurotransmitters is the fact that there appears to be a higher degree of inconsistency in the examined literature than that observed in genetic and brain structure research. For example, studies into the relationship between serotonin and aggression have produced only mixed results, with some offering support for a negative correlation (as cited by Ferguson & Beaver, 2009), while others point in the direction of a positive one (Carrillo et al., 2009; Van de Giessen et al., 2014). It has been suggested that aggression type (reactive vs. proactive) is likely responsible for this inconsistency (Blair, 2006; Carrillo et al., 2009; Van de Giessen et al., 2014). This proposed distinction underscores the intricacy of characteristics underlying psychopathy and its many risk factors. Similarly, the distinction between “primary” and “secondary” psychopathy, which has thus been little investigated in this context (Goulter et al., 2019; Sethi et al., 2018), may explain contradictory findings.

In addition to specific distinctions, determining the role individual neurotransmitters play in psychopathy is convoluted by the notion that some appear to have a differing, synergistic-like effect when interacting with one another. This is evident in the reported interactive effect of dopamine and epinephrine in producing both increased aggression and reduced empathy (Antelman & Caggiula, 1977; Cases et al., 1995; Thomas & Palmiter, 1997); these qualities were not observed when epinephrine was examined in isolation (Gerra et al., 2003). Furthermore, an interaction between cerebrospinal fluid dopaminergic and serotonergic metabolite levels has been speculated to underlie aggression (Soderstrom et al., 2003), but this interaction has been rarely studied, while studies frequently examined serotonin alone (see Carrillo et al., 2009).

Because many studies have such a narrow scope, bridging the gap between their results becomes a difficult task. Moving beyond general observations of suppression, in a direction that leads to a fuller understanding of the interplay between all of the explored evidence, would presently be unfeasible. The inconsistency in findings appears to be rooted in a number of factors including variability in conceptualizations and measurements used across studies (Kim-Cohen et al., 2006; Lorber, 2004); absent consideration of environmental experiences that interact with one’s genotype, and their epigenetic outcomes (Fowler et al., 2009); and the use of broad categories and classifications for traits and behaviors that may, in fact, have different underlying neurobiological characteristics (e.g., measuring aggression without accounting for aggression types, as discussed earlier; Carrillo et al., 2009; Van de Gissen et al., 2014). Similar arguments may be made based on emergent research into primary and secondary variants of psychopathy (Goulter et al., 2019; Kimonis et al., 2017; Sethi et al., 2018). It is probable that other distinctions and interactions have yet to be uncovered; and until they are, understanding the relationship between genes, neurotransmission, brain activity, and psychophysiology in psychopathy remains obscured for the most part.

## 10. Neurobiology and development: directions for interdisciplinary collaboration

Considering the potential for early-life epigenetic change and early evolution of neurobiological differences produced by stress and adversity, it is critical that longitudinal perspectives be taken in psychopathy research utilizing neurobiological measures. In fact, cross-sectional designs using 7-year-olds may offer little added benefit to using adult participants, considering some changes are likely to occur in the first 3 years of life. In contrast, given reliance on incarcerated samples, much of the extant research excludes individuals who have entered and passed midlife, hindering predictions about stability of psychopathy traits over time. Furthermore, any observations made during adulthood are best interpreted as snapshots in time, which cannot inform our understanding of etiology nor prognosis, given later-in-life biological and behavioral changes (e.g., Hawes et al., 2014; Vitacco & Vincent, 2006). For instance, psychopathy reportedly increases in adolescence and diminishes as individuals reach young adulthood (Salekin, Rosenbaum, Lee, & Lester, 2009). Similarly, psychopathy scores (and offending behaviors) are generally significantly reduced as previously labeled ex-inmates enter into their 60s (Putkonen et al., 2010), suggesting an instability in psychopathy over the life course.

Moreover, the current understanding of genetic and neurobiological psychopathic mechanisms is based primarily on observations of incarcerated men of European descent. This limitation is particularly problematic to genetic research, since different ethnic groups vary in genetic manifestation of various conditions (Burchard et al., 2003), including psychopathy (Lu et al., 2003) and its socio-cultural interpretation (Mokros et al., 2011). Multicultural investigations can be uniquely informative, since non-invariance in the psychopathy construct between samples from similar cultural backgrounds (i.e., North Americans and Germans) has been noted (Mokros et al., 2011). Research using community samples, women, and people of non-European heritage is necessary before any sweeping etiological conclusions can be drawn.

Also critical to consider are the ways constructs are measured across current research. Psychopathy is measured in a wide variety of ways, with some measures relying heavily on aggression and criminal justice involvement. Moreover, reliance on criminal history in the measurement of psychopathy is particularly problematic given Yang et al.’s (2010) findings that some physiological differences in individuals with arrest histories did not appear in those without such histories. Similarly, adversity-exposure measurement is often problematic in that different instruments capture different adverse events and all exclude adversity that occurs during the first 3 years of life. Furthermore, many measures do not assess the most invisible form of maltreatment: deprivation and neglect (Perry, 2002). Though an inherently challenging endeavor, researchers should go beyond self-report questionnaires onto developing and utilizing measures that assess early-life experience holistically.

### 10.1. Limitations

In the current review, authors sought to summarize and evaluate the state of knowledge on the etiology of psychopathic traits, by focusing attention on research into genetic, neuroanatomical, and neurotransmission activity correlates of psychopathic and antisocial traits. Inevitably, such a pursuit is fraught with challenges related to the differing conceptualizations and measurements of the psychopathy construct. While a broad view of psychopathic personality is useful for a first effort, it should likely be preceded with more narrow reviews and meta-analytic efforts. Yet it is uncertain that these, too, would yield a clearer answer for the question at hand. For instance, a review of a subset of studies using PCL instruments (46% of the studies included in this review) did not produce increased consistency of findings regarding genetic or neurobiological mechanisms. That is, utilizing Hare’s (2003) definition of psychopathy (to the exclusion of others) did not appear to lead to a better overall understanding of its etiology. It is possible that additional research exists that has not been included in this review (e.g., because it is yet to be published, or because it was unavailable at the time), and which may add support for some of the more pronounced findings. Furthermore, no statistical analysis was performed for the purpose of this review, and thus, it is possible that some findings, while inconsistent across studies, are nonetheless meaningful in the samples in which they were observed, or across several of these samples. Meta-analytic strategies to evaluate subsets of these findings (e.g., regarding genetic influences) can shed further light on these questions.

### 10.2. Implications and conclusions

While it is important to consider the current status of knowledge and ways to advance research into psychopathic traits, it is equally important that an integrated perspective inform current practices in management and treatment of psychopathic individuals. Specifically, clinicians and legal professionals should consider how yet-unsubstantiated theories about its etiology have affected both prognosis and treatment of psychopathy. It is likely that, in the absence of a conceptualization of psychopathy as a biological malady, clinicians would perceive both psychopathy treatment and its desired outcomes differently. This is particularly important since the belief in its innate, immutable nature has affected the use of the psychopathy construct in high-stakes legal situations (e.g., death penalty sentencing hearings; Cox et al., 2016), with overwhelmingly negative outcomes.

**Prognosis.** Based on anecdotal evidence from case studies such as Cleckley’s (1941), along with hereditary research, psychopathy has largely been perceived as immutable and untreatable (Looman, Abracen, Serin, & Marquis, 2005; Skeem, Monahan, & Mulvey, 2002). Yet research demonstrates that, even without treatment, psychopathic traits and temperamental qualities are not stable over time (Hawes et al., 2014; Hopwood et al., 2013; Josefsson et al., 2013; Kandler, Riemann, & Angleitner, 2013; Laceulle, Ormel, Vollebergh, Aken, & Nederhof, 2014). Even in actuarial recidivism risk assessment, considerations for the passage of time and aging have been recommended (Harris & Rice, 2007). Given associations between psychopathy and early-life stress, it is notable that the idea of change has been even more pronounced across traumatic stress literature. Perry (2002) found that in following chronically neglected children over time, recovery of brain structure and function over a year period was inversely proportional to age of removal from the neglectful environment, noting that even the oldest children made significant gains over time. Is it then possible that psychopathy is not as immutable as many believe? To assess this, it is worthwhile to consider common treatment strategies.

**Treatment.** Many of the treatment paradigms targeting psychopathy are based in correctional settings, with the expressed goal of increasing remorse and reducing criminal or violent behavior (Harris & Rice, 2006; Looman et al., 2005). Yet few clinicians would recommend a similar treatment for trauma-affected populations; aggressive or violent behavior in this context is typically seen as one of many traumatic stress symptoms to be addressed in treatment, and self-reproachfulness is often explicitly discouraged (Ford, Chapman, Connor, & Cruise, 2012). Further, trauma treatment commonly involves characteristics that are diametrically opposed to those seen in offender treatment. If clinicians treating individuals with psychopathic traits were to borrow from the complex trauma literature (e.g., Perry, 2009), they would recognize the possibility that many attempts at psychopathy treatment have not worked because of their failure to include the necessary elements for neurodevelopmental change: willing participation in consistent, patterned interaction with a caring, trusted person.

## 11. Conclusion

For several decades, research has focused intensely on identifying the biological underpinnings of psychopathy, under the assumption that this construct represented a unification between life-course stable, immutable traits. Yet despite the passage of much time, a significant investment, and substantial technological advances in measurements of biological differences, few consistent conclusions can be made from this research. Indeed, some differences in neurological structure and activation patterns have been observed, but these leave the research community no closer to identifying an etiology for the construct. Practitioners, nonetheless, have eagerly adopted a biologically deterministic perspective on psychopathy, utilizing research measures to determine the fates of individuals accused of crimes (including children; for an example see Rockett, Murrie, & Boccaccini, 2007), and to make decisions regarding treatment procedures and outcomes (Archer, Buffington-Vollum, Stredny, & Handel, 2006; Odgers, Reppucci, & Moretti, 2005; Salekin, 2002). This, in turn, could lead more individuals to experience the kind of treatment that may only serve to perpetuate the lack of remorse, empathy, and human connection presumed to underlie psychopathy. Ultimately, even the staunchest supporters of the biological hypothesis can agree that environmental influences cannot be excluded from a complete understanding of psychopathy. It is critical that in both research and practice, the influence of environment be evaluated alongside other factors affecting the development of psychopathic traits. Hence, future research should provide potential to evaluate neurodevelopmental change (whether through epigenetic mechanisms or neural plasticity) and predictors thereof, since these will likely serve to inform both prevention and intervention efforts targeting psychopathic individuals.
